# Combining statistical shape modeling, CFD, and meta‐modeling to approximate the patient‐specific pressure‐drop across the aortic valve in real‐time

**DOI:** 10.1002/cnm.3387

**Published:** 2020-09-13

**Authors:** M. J. M. M. Hoeijmakers, I. Waechter‐Stehle, J. Weese, F. N. Van de Vosse

**Affiliations:** ^1^ Department of Biomedical Engineering Eindhoven University of Technology Eindhoven The Netherlands; ^2^ ANSYS Inc Villeurbanne France; ^3^ Philips Research Hamburg Germany

**Keywords:** aortic valve stenosis, computational fluid dynamics, heart valve disease, meta‐modeling, statistical shape modeling

## Abstract

**Background:**

Advances in medical imaging, segmentation techniques, and high performance computing have stimulated the use of complex, patient‐specific, three‐dimensional Computational Fluid Dynamics (CFD) simulations. Patient‐specific, CFD‐compatible geometries of the aortic valve are readily obtained. CFD can then be used to obtain the patient‐specific pressure‐flow relationship of the aortic valve. However, such CFD simulations are computationally expensive, and real‐time alternatives are desired.

**Aim:**

The aim of this work is to evaluate the performance of a meta‐model with respect to high‐fidelity, three‐dimensional CFD simulations of the aortic valve.

**Methods:**

Principal component analysis was used to build a statistical shape model (SSM) from a population of 74 iso‐topological meshes of the aortic valve. Synthetic meshes were created with the SSM, and steady‐state CFD simulations at flow‐rates between 50 and 650 mL/s were performed to build a meta‐model. The meta‐model related the statistical shape variance, and flow‐rate to the pressure‐drop.

**Results:**

Even though the first three shape modes account for only 46% of shape variance, the features relevant for the pressure‐drop seem to be captured. The three‐mode shape‐model approximates the pressure‐drop with an average error of 8.8% to 10.6% for aortic valves with a geometric orifice area below 150 mm^2^. The proposed methodology was least accurate for aortic valve areas above 150 mm^2^. Further reduction to a meta‐model introduces an additional 3% error.

**Conclusions:**

Statistical shape modeling can be used to capture shape variation of the aortic valve. Meta‐models trained by SSM‐based CFD simulations can provide an estimate of the pressure‐flow relationship in real‐time.

## Graphical abstract

In this study, statistical shape modeling, computational fluid dynamics (CFD), and meta‐modeling techniques were combined to obtain a cheap‐to‐evaluate meta‐model. The meta‐model relates shape variation of 74 segmented aortic valves, to variations in CFD‐computed flow vs pressure‐drop curves. Once trained, meta‐models can be a cheap and robust alternative to compute intensive CFD simulations.

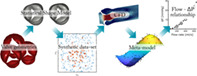

## 1 INTRODUCTION

Advances in medical imaging and image segmentation techniques have resulted in a tremendous increase in the use of complex three‐dimensional patient‐specific simulations over the last two decades.^1^ The availability and applicability of complex three‐dimensional computational models for clinical applications is further stimulated by high performance computing and development of more robust and efficient codes to solve the governing equations. Computational models are now widely adapted throughout the cardiovascular research community. Models are used, for example, to assess mechanical^2^, ^3^ or hemodynamic^4^, ^5^, ^6^, ^7^, ^8^ quantities such as stress, wall shear stress, or pressure drops.

Imaging modalities such as Computed Tomography, Magnetic Resonance Imaging, and ultrasound are used on a daily basis in the clinic. With segmentation tools, patient‐specific geometries are readily obtained from the acquired imaging data. These geometries can then be used as input to computational fluid dynamics (CFD) simulations to provide detailed pressure and velocity fields in the blood vessel. These hemodynamic quantities are difficult, or sometimes impossible to assess with imaging techniques alone. Patient‐specific CFD simulations have already proven their diagnostic value for coronary disease.^4^, ^6^ However, in the field of heart‐valve disease, CFD is not yet accepted for clinical diagnostics. Instead, three‐dimensional CFD simulations are primarily used to understand the fundamental principles of valve dynamics,^9^ left ventricular hemodynamics,^10^ or for valve design.^11^

It is not without reason that heart valve CFD simulations are not yet used for clinical decision making or diagnostics. Valve simulations are particularly challenging from a numerical and imaging perspective. To adequately model valve hemodynamics throughout the entire cardiac cycle, coupled fluid‐structure interaction simulations are required. These simulations are difficult due to the large deformations of the valve leaflets, and consequently of the computational grid. Large grid deformations make traditional Arbitrary Euler‐Lagrange less robust (convergence and mesh deformation) and efficient (re‐meshing). To address these difficulties, other numerical schemes, such as immersed‐boundary or adaptive cut‐cell methods are generally used. These numerical methods are more efficient, at the expense of solution accuracy at the fluid‐solid interface. However, even with these methods, the computational cost for such simulations is in the range of days to weeks.^12^ Hence, fluid‐structure interaction simulations become intractable for day‐to‐day clinical practice. Alternatively, simplified fixed‐grid CFD models can be used to obtain a reasonable approximation of fluid flow through, and around the aortic valve.^13^ Indeed, the valve opens and closes very rapidly, and may not substantially influence flow patterns at peak systole. This is supported by Astorino et al, who showed that a simplified fixed grid 3‐D CFD model of the aortic valve yields similar results at peak‐systole as those obtained from fluid‐structure interaction simulations.^14^ Even though simplified, such models may already provide valuable spatial and/or temporal hemodynamic information to clinicians. Moreover, these models are computationally cheap, more robust, and enable the development of automated simulation frameworks that are suitable for clinical practice.

Besides the numerical challenges, patient‐specific three‐dimensional geometrical models of the valves are difficult to obtain from imaging data. Automatic segmentation frameworks often struggle with the complex, three‐dimensional, and thin nature of the leaflets. Many authors rely on semi‐automatic or manual segmentation to obtain the three‐dimensional geometry of the valve.^7^ A small number of authors developed automated segmentation frameworks that enable (aortic) valve segmentation.^15^, ^16^, ^17^ These segmentation frameworks make use of parameterized geometries^17^ or deformable models^15^, ^16^, ^18^ that are adapted to the patient's imaging data.

The deformable‐model‐based segmentation approach proposed by Weese et al yields a structured surface mesh with consistent inter‐patient topology, that is, a triangulated surface with a consistent number of faces and vertices.^15^ Consistent inter‐patient topology of the segmentation mesh enables Statistical Shape Modeling (SSM).^19^, ^20^ SSM utilizes Principal Component Analysis to extract the main directions of (geometrical) variance, interpreted as shape modes. Any patient‐specific mesh can then be reconstructed by the mean mesh, and a weighted combination of a small number of shape modes. The weights can be regarded as parameters that define the geometry. This parametric description can then be used to obtain an approximate reconstruction of any mesh, within or outside of the training set. Alternatively, the SSM can be used to generate synthetic meshes that are representative of the training set.

The SSM describes the variation of shape with just a few parameters. This feature can be combined with CFD simulations to explore the relationship between shape and simulation outcome. Moreover, such a simulation framework can generate enough data to train meta‐models. Meta‐models typically rely on regression and/or interpolation of a set of learning points to find a relationship between model input parameters and output parameters. The continuous function describing this relationship can be an efficient surrogate for the high fidelity, but computationally costly, simulations. Meta‐models are widely used for design optimization,^21^ uncertainty quantification, and sensitivity analysis.^22^ These meta‐models can be constructed with various methods, such as: polynomials,^23^ radial basis functions,^24^ and Kriging.^25^ Since meta‐model selection is often difficult, weighted meta‐model ensembles—and the automatic selection thereof—were developed.^26^, ^27^

The aim of this paper is to evaluate the performance of a meta‐model with respect to high‐fidelity CFD simulations. To achieve this, the aortic valve was parameterized by making use of a SSM. With the resulting parametric model of the aortic valve, a large number of training samples were generated. Consequently, CFD simulations were launched, and the meta‐model was trained on the SSM parameters, and the CFD results. Finally, the meta‐model and CFD simulations of the reconstructed geometries are compared to the output of the CFD simulations of the segmented mesh to evaluate the quality of both the SSM and meta‐model.

## 2 METHODS

### 2.1 Imaging data

In this manuscript, Computed Tomography imaging datasets of 74 patients were available. From these 74 datasets, 12 were provided by three clinical centers: the Sheffield Teaching Hospital NHS Foundation Trust; the Catharina Hospital in Eindhoven; and Deutsches Herzzentrum as part of the EurValve research project. Furthermore, a retrospective data‐set of 62 Computed Tomography images was available.^15^, ^16^, ^28^ Images were acquired with an in‐plane spatial resolution of 0.31 to 0.68 mm, and slice thickness of 0.34 to 0.70 mm. Images were acquired with Electrocardiography gated Computed Tomography, and segmentations represent the peak‐systolic state of the aortic valve.

### 2.2 Aortic valve segmentation

**FIGURE 1:**
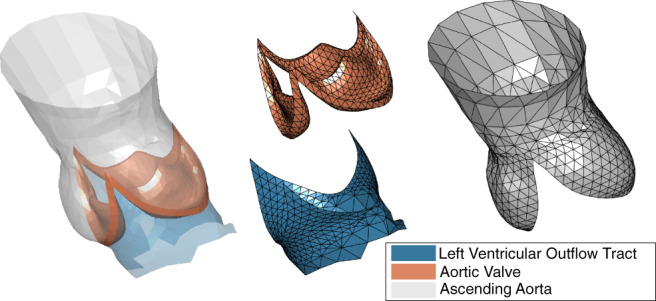
Typical example of a segmentation mesh of the aortic valve region, the output of the Shape Constrained Deformable Model framework.^15^, ^16^ In blue: the left ventricular outflow tract. In red: the aortic valve. In gray: the aortic root

Segmentation of the aortic valve was performed with a Shape Constrained Deformable model framework, presented in earlier work.^15^, ^16^, ^28^ In the segmentation framework, a template mesh model is iteratively adapted to an image. From the resulting segmentation mesh, a submesh (ℳ) was extracted for this study (Figure 1). The submesh ℳ contains the left ventricular outflow tract, the aortic valve, and (part of) the aortic root (Figure 1). ℳ has a consistent number of vertices *k*, and consistent topology (T), resulting in mesh correspondence between patients. Each surface mesh is defined by *k* = 1808 vertices, and 4223 triangular faces.

### 2.3 Statistical shape modeling

The SSM describes the training set by a mean shape and the main modes of (shape) variation. SSM's are widely described and applied in literature, and for a comprehensive overview of its applications the authors refer to a review by Heimann and Meinzer^19^—or more recently—Biglino and colleagues.^29^ Following segmentation, a structured surface mesh ℳ, representing the aortic valve, is available for each patient.

(1) $$ℳ=ℳ\left(x,T\right)$$

With **x** the coordinate vector of the vertices and Tthe topology of the mesh. For the SSM, segmentation meshes were aligned by a generalized Procrustes analysis,^30^ which optimally translated, rotated, and scaled each of the meshes by minimizing the sum of squared errors. Following alignment, the coordinates of *k* = 1808 vertices were concatenated into a single vector (column) of length 3*k* for each segmentation *i*:

(2) $$x_{i}={\left[x_{1},y_{1},z_{1},x_{2},y_{2},z_{2},\ldots x_{k},y_{k},z_{k}\right]}^{T}$$

Averaging over *N*_*s*_ segmentations, the mean shape was obtained:

(3) $$\bar{x}=\frac{1}{N_{s}}\sum_{i=1}^{N_{s}}x_{i}$$

Using **x**_*i*_ and $\bar{x}$ the covariance matrix S with size 3*k* × 3*k* was computed by:

(4) $$S=\frac{1}{N_{s}-1}\sum_{i=1}^{N_{s}}\left(x_{i}-\bar{x}\right){\left(x_{i}-\bar{x}\right)}^{T}$$

Consequently, the eigendecomposition of S yielded the principal modes of variation, that is, the eigenvectors ***ϕ***_*m*_, and corresponding eigenvalues (*λ*_*m*_). *λ*_*m*_ and ***ϕ***_*m*_ were ordered from high to low explained variance. In the three‐dimensional case the eigenvectors ***ϕ***_*m*_ also have length 3*k*. The eigenvectors represent the principal directions of variation, and are referred to as shape modes in the context of shape modeling. Any shape **x**_*i*_ can then be reconstructed (${\hat{x}}_{i}$) by a linear combination of *N*_*m*_ shape modes weighted by coefficients *α*_*i*, *m*_.

(5) $${\hat{x}}_{i}=\bar{x}+\sum_{m=1}^{N_{m}}\alpha_{i,m}\phi_{m}\;i\in \left\{1,2,\ldots N_{s}\right\}$$

Note that ${\hat{x}}_{i}$ in Equation (5) is an approximation of **x**_**i**_. The shape coefficients *α*_*m*_ are patient‐specific, and were obtained by the dot product of the centered data and the eigenvectors:

(6) $$\alpha_{i,m}=\phi_{m}^{T}\cdot \left(x_{i}-\bar{x}\right)$$

where *α*_*i*, *m*_ is contained in the coefficient vector ***α***_*i*_:

(7) $$\alpha_{i}=\left\{\alpha_{i,m}:m=1,\ldots ,N_{m}\right\}$$

In fact, Equation (6) is equal to minimizing the ℓ_2_ norm of the difference between the reconstruction and the original:

(8) $$\min_{\alpha_{i}\in R^{N_{m}}}\left({\left\|{\hat{x}}_{i}\left(\alpha_{i}\right)-x_{i}\right\|}_{2}\right)$$

The minimization of Equation (8) was manipulated with a weight vector **w** (1 × 3*k*) to increase the weight of regions that are physically more relevant:

(9) $$\min_{\alpha_{i}\in R^{N_{m}}}\left({\left\|\left({\hat{x}}_{i}\left(\alpha_{i}\right)-x_{i}\right)\cdot w\right\|}_{2}\right)$$

**FIGURE 2:**
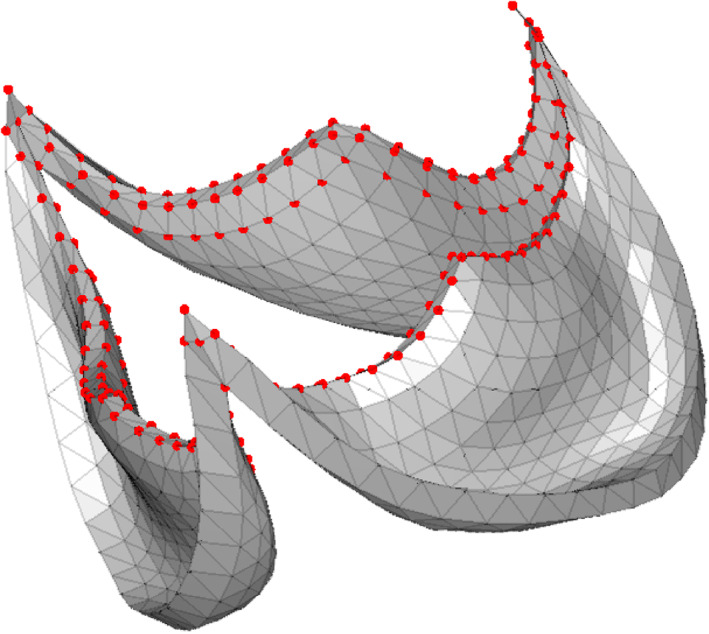
Vertices that are part of and that are adjacent to the free cusp edge are highlighted. Highlighted vertices were assigned a weighting factor of five. All other vertices were given a weight factor of 1

The introduction of **w** allowed to control the weight of each vertex to the minimization problem. When computing pressure drops, the opening area of the valve is physically most relevant. Consequently, a low reconstruction error for the vertices that were part of and adjacent to the free cusp edges of the valve was desirable. **w** was empirically established, and in this study weighting the 216 vertices that were part of or adjacent to the free cusp edges (Figure 2) five times stronger than other vertices gave good results. Note that Equation (9) is the same as Equation (6) when $w=\vec{1}$. For each segmentation sample, coefficients of ***α*** were found by minimizing Equation (9).

Subsequently, the original vertices **x** from any segmented mesh ℳ (see Equation (1)) were replaced by $\hat{x}$ to yield an approximate reconstruction ℳ_*r*_ of the segmentation:

(10) $$ℳ\left(x,T\right)\;\approx \;{ℳ}_{r}\left(\hat{x},T\right).$$

#### 2.3.1 Statistical shape model performance

In literature, SSM performance is typically evaluated with compactness and generalizability metrics.^31^ Compactness is a function of the number of modes, and defined as the sum of variances, normalized by the cumulative variance:

(11) $$C\left(N_{m}\right)=\frac{\sum_{m=1}^{N_{m}}\;\lambda_{m}}{\sum_{m=1}^{N_{t}-1}\;\lambda_{m}}$$

where *N*_*t*_ is the number of samples used to train the SSM.

To test how well the model generalizes to unseen data (generalization ability), the average sum of squared errors of a leave‐one‐out procedure was computed:

(12) $$G\left(N_{m}\right)=\frac{1}{N_{t}}\sum_{i=1}^{N_{t}}{\left\|x_{i}-{\hat{x}}_{i}\left(N_{m}\right)\right\|}^{2}$$

where **x**_*i*_ is the left‐out shape, and ${\hat{x}}_{i}\left(N_{m}\right)$ the approximated shape. The approximated shape was obtained by using *N*_*m*_ number of shape modes from a SSM that was trained on all samples, but excluding the sample to be approximated.

In addition, three physically relevant areas were quantified from the mesh to judge reconstruction performance, the area of the left ventricular outflow tract, aortic valve, and ascending aorta. In this manuscript, the aortic valve area refers to the geometric orifice area, and is not to be confused with the effective orifice area, the metric used in the clinic.

### 2.4 Computational fluid dynamics

**FIGURE 3:**
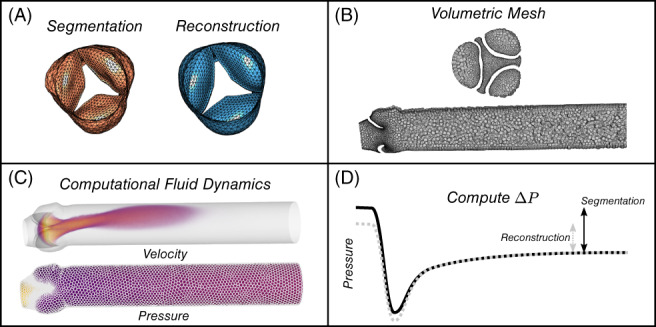
Graphical representation of the workflow from segmented and reconstructed surface meshes A). B) From the surface meshes a volumetric mesh was created. C) Volumetric meshes were used in the CFD simulations. D) Consequently, the recovered pressure‐drop is extracted from the CFD simulations

Sections 2.2 and 2.3 describe how the segmentation meshes ℳ and the reconstructed meshes ℳ_*r*_ were obtained. The volume enclosed by these surface meshes were discretized (meshed) to enable CFD simulations. To achieve this, first the outflow boundary of ℳ and ℳ_*r*_ were extruded by 10 times the radius of the sinotubular junction. The inflow boundary normal was defined by three vertices at the valve annulus. This plane was moved proximal such that it was in the most proximal position, while still maintaining an enclosed surface. Finally, the shrink‐wrapping option in ANSYS Fluent Meshing 18.2 (ANSYS Inc., Canonsburg, Pennsylvania) was used to automatically generate a volumetric mesh with approximately 2 ⋅ 10^5^ to 3 ⋅ 10^5^ polyhedral elements (Figure 3). Edge lengths of the polyhedrals were chosen based on a mesh‐sensitivity study, and ranged between 0.15 and 2.0 mm. As part of a mesh refinement study, maximum element sizes were reduced to 1.0 and 0.5 mm respectively, which typically improved the results by less than 1%. Hence, a maximum size of 2.0 mm provided a good trade‐off between accuracy and computational load.

Blood was modeled as an incompressible Newtonian fluid with a density of 1060kg/m^3^ and dynamic viscosity of 0.004 Pa ⋅ s. For the segmentation and reconstructed meshes, steady‐state simulations were performed at flow rates of 200, 300, 400, 500, and 600 mL/s, and were used to approximate the pressure‐drop flow relationship for each patient. At the inflow boundary a plug‐velocity profile was prescribed. Pressure at the outlet was set to zero, and no‐slip boundary conditions were assumed at the walls.

Areas of the left ventricular outflow tract (the inflow‐boundary) were between 2.4 and 6.4 cm^2^. With blood‐like fluid properties, flow‐rates, and geometric dimensions, inlet Reynolds numbers for the CFD simulations were estimated to be between 2400 and 12 000. Hence, a Shear Stress Transport *k* − *ω* model, with 5% turbulence intensity at the inlet, was used to model turbulence.^32^ The output variable of interest is the net pressure‐drop across the aortic valve, and defined as the pressure at the inflow boundary minus the downstream recovered pressure (Figure 3D). For convenience, the downstream pressure was taken at the outflow boundary, which is a reasonable assumption when pressure‐loss in the straight extended section is negligible with respect to pressure‐loss due to the presence of the valve. Convergence was assumed when the pressure drop did not change anymore. Small oscillations in the solution were observed. Hence, an average pressure residual *R*_*p*_ was defined to monitor convergence.

(13) $$R_{p}\left(j\right)=\frac{|{\bar{p}}_{j}-{\bar{p}}_{j-200}|}{{\bar{p}}_{j}}\leq 0.001$$

Here ${\bar{p}}_{j}$ is the area‐weighted pressure at the inlet boundary at iteration *j*, averaged over the last 200 iterations. ${\bar{p}}_{j-200}$ is then the area‐weighted pressure at the inlet, averaged over the range [*i* − 400, *i* − 200].

Out of the total of 2960 simulations that were performed (74 segmentations, 8 meshes, that is, 7 reconstructions and 1 segmentation, and 5 flow rates), 81% exhibited maximum relative deviations of <1% with respect to the iteration‐averaged mean (${\bar{p}}_{j}$), and 99% exhibited maximum relative deviations below 5%. Only 11 (0.4%) simulations experienced relative deviations from the mean larger than 10%, but all of these yielded negligible iteration‐averaged pressure‐drops (<0.5 mmHg).

The governing equations were solved with ANSYS Fluent 18.2 (ANSYS Inc., Canonsburg, Pennsylvania), a finite volume based CFD solver. Simulations were performed on the ACC Cyfronet AGH Prometheus supercomputer (Academic Computer Centre Cyfronet, AGH University of Science and Technology, Kraków, Poland). Each CFD simulation was assigned four CPU's and took between 10 and 30 minutes to complete.

### 2.5 Meta‐model training

#### 2.5.1 Design of experiments

Statistical shape modeling allowed us to describe geometrical changes with a limited number of parameters. These parameters were used to build a meta‐model in parameter space. The parameter space was restricted to five parameters: the first three shape coefficients (*α*_1_, *α*_2_, *α*_3_) of the SSM trained on all available segmentations, a global scaling parameter *s*, and flow rate *Q* (Table 1). The three shape coefficients were used to generate synthetic meshes throughout the input space, and were constrained to lie within $[-3\sqrt{\lambda_{m}},3\sqrt{\lambda_{m}]}$. Feasible limits for the global mesh scaling parameter were determined from the reconstruction procedure. Flow‐rates between 50 and 650 mL/s were considered. Upper and lower limits of the individual parameters are described in Table 1. The input‐space was sampled with Latin Hypercube designs of 25, 50, 100, 200, 400, 800, and 1600 samples. Learning points were excluded from meta‐model training when the aortic valve opening was less than 20 mm^2^, when the pressure drop exceeded 300 mmHg, or when the simulations diverged.

**TABLE 1:** Input parameters and their respective limits

Description	Symbol	Unit	Minimum	Maximum
Valve opening	*α*_1_	‐	$-3\sqrt{\lambda_{1}}$	$3\sqrt{\lambda_{1}}$
	*α*_2_	‐	$-3\sqrt{\lambda_{2}}$	$3\sqrt{\lambda_{2}}$
	*α*_3_	‐	$-3\sqrt{\lambda_{3}}$	$3\sqrt{\lambda_{3}}$
Global mesh scaling	*s*	‐	0.8	1.25
Flow‐rate	*Q*	ml/s	50	650

#### 2.5.2 Meta‐model methodology

Selecting the most suitable meta‐model, and meta‐model settings is often difficult since no universal meta‐model exists that performs well for all problems. Hence, several authors worked on ensemble approaches, where a weighted sum of meta‐models is considered.^26^, ^27^ One such algorithm is the Genetic‐Aggregation meta‐model, available in the commercial package ANSYS DesignXplorer (ANSYS Inc., Canonsburg, Pennsylvania). In this algorithm meta‐model selection is automated by minimizing a penalized predictive score.^27^ In this study, the Genetic‐Aggregation meta‐model was trained with the available simulation data. A comprehensive overview of the advantages and shortcomings of such a ensemble‐type meta‐model approach is beyond the scope of this work. But to give the reader a basic understanding of the working principle, a short summary follows.

The goal of a ensemble‐type meta‐model is to obtain the best weighted‐average of a selection of meta‐models:

(14) $${\hat{y}}_{\mathrm{ens}}\left(x\right)=\sum_{i=1}^{N_{e}}\beta_{i}\cdot {\hat{y}}_{i}\left(x\right)$$

where ${\hat{y}}_{\mathrm{ens}}$ is the prediction at *x* of the final meta‐model, that is, the weighted ensemble of various meta‐models and their settings. ${\hat{y}}_{i}$ is the prediction at *x* of the *i*th meta‐model, weighted by *β*_*i*_. And *N*_*e*_ is the number of meta‐models used. The idea is that appropriate weighting of the individual meta‐models will cancel out errors in the prediction of the individual meta‐model. The trick is then to find the weights that gives the best quality ensemble prediction ${\hat{y}}_{\mathrm{ens}}$. However, assessing optimal quality objectively is not straightforward. In the Genetic‐Aggregation algorithm, a penalized predictive score ℒ is introduced.^27^ This score combines three components (Equation (15)): (a) optimizing the internal accuracy by evaluating the mean square error on training samples/points; (b) use a 10‐fold cross‐validation to evaluate predictive capability on unseen samples; and (c) minimize over‐fitting of the meta‐model by a thin‐plate spline Bending Energy Functional.^27^, ^33^ The penalized predictive score is then constructed by weighting the contribution of each of these components:

(15) $$ℒ\left({\hat{y}}_{\mathrm{ens}}\right)=\underset{a}{\underset{︸}{\gamma_{1}ℛ\left({\hat{y}}_{\mathrm{ens}}\right)}}+\underset{b}{\underset{︸}{\gamma_{2}{ℛ}_{10\mathrm{CV}}\left({\hat{y}}_{\mathrm{ens}}\right)}}+\underset{c}{\underset{︸}{\gamma_{3}E\left({\hat{y}}_{\mathrm{ens}}\right)}}$$

This loss function ℒ is defined for the aggregate meta‐model ${\hat{y}}_{\mathrm{ens}}$ on the set of training points. *γ*_1_, *γ*_2_, and *γ*_3_ are constants, and kept at 1, 0.5, and 0.25 respectively. The optimal aggregation of meta‐models is then obtained by minimizing ℒ under the condition that

(16) $$\sum_{i=1}^{N_{e}}\beta_{i}=1$$

This problem was shown to be quadratic and has an analytic solution.^27^ In this work, we used the algorithm PPS‐OS described in Ben Salem and Tomaso^27^ where 32 meta‐model candidates of four types are considered: 24 Kriging, 3 polynomial regression, 2 support vector machine, and 3 moving least squares.

For a more comprehensive overview of such ensemble‐type meta‐models, or the Genetic‐Aggregation methodology specifically, the reader is encouraged to consult the work by Viana et al,^34^ Acar,^26^ or Ben Salem and Tomaso.^27^

#### 2.5.3 Evaluating approximation error

Approximate reconstruction of the segmentation mesh (Equation 5) inherently introduces geometrical errors which will affect the computed pressure‐drop. Moreover, the meta‐model is a further simplification that approximates the simulated pressure‐drop. To evaluate the influence of both approximations on the pressure‐drop, differences in the pressure‐drop results were expressed as the root mean square error (RMSE—Equation 17), relative RMSE (Equation 18), and mean absolute percent error (MAPE—Equation 19):

(17) $${ɛ}_{\mathrm{RMS}}=\sqrt{\frac{1}{N}\sum_{i=1}^{N}{\left(\Delta {\hat{p}}_{i}-\Delta p_{i}\right)}^{2}}$$

(18) $${ɛ}_{\text{rRMS}}=\sqrt{\frac{1}{N}\sum_{i=1}^{N}{\left(\frac{\Delta {\hat{p}}_{i}-\Delta p_{i}}{\Delta p_{i}}\right)}^{2}}$$

(19) $${ɛ}_{\mathrm{MAP}}=\frac{1}{N}\sum_{i=1}^{N}|\frac{\Delta {\hat{p}}_{i}-\Delta p_{i}}{\Delta p_{i}}|$$

Here, $\Delta {\hat{p}}_{i}$ is the approximated pressure‐drop from the reconstructed mesh or meta‐model, and Δ*p*_*i*_ the pressure‐drop computed from the CFD model. *N* represents the total number of available samples. The total number of available samples for a comparison between the segmented model and the reconstructed model is 370 (74 segmentations times 5 flow rates).

## 3 RESULTS

### 3.1 Training‐set characteristics

**FIGURE 4:**
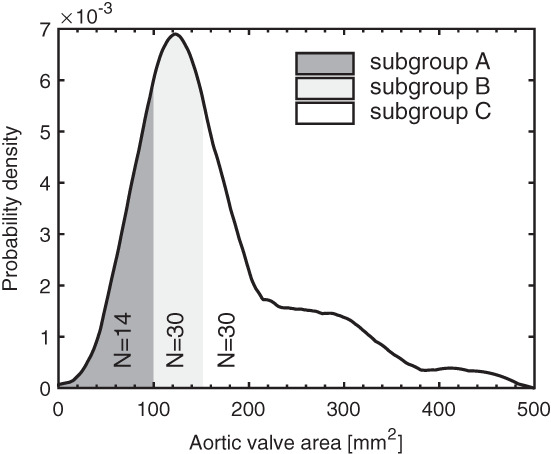
Distribution of the aortic valve area in the complete training set of all 74 segmentations. Dark gray: subgroup A, cases with a geometric valve area below 100 mm^2^. Light gray: subgroup B, cases with a valve area between 100 and 150 mm^2^. White: subgroup C, valve area is larger than 150 mm^2^

Aortic valve segmentations were split into three groups based on their projected geometric aortic valve orifice area and the recommendations from the European Association of Cardiovascular Imaging and the American Society of Echocardiography.^35^ Threshold criteria for these groups were: severe—subgroup A: valve area below 100 mm^2^; intermediate—subgroup B: valve area between 100 and 150 mm^2^, and mild/healthy—subgroup C: valve area larger than 150 mm^2^. Of all 74 aortic valve segmentations, 14 were in subgroup A (severe), 30 in subgroup B (intermediate), and 30 in subgroup C (mild/healthy). Figure 4 demonstrates how the aortic valve areas were distributed over the 74 segmentations, with a median of 143 mm^2^, and mean of 166 mm^2^. Cases with a valve area over 150 mm^2^ were distributed over a wide range with a maximum of 440 mm^2^. The smallest valve area in subgroup A was 46 mm^2^.

### 3.2 Statistical and geometrical performance of the shape model

Segmentations were reconstructed with seven models, each using more shape modes for reconstruction (*N*_*m*_ = {0,1,2,3,4,5,20}). Shape coefficients for each respective model were found by Equation (9). The following section demonstrates how the number of shape modes affect geometrical performance of the SSM.

**FIGURE 5:**
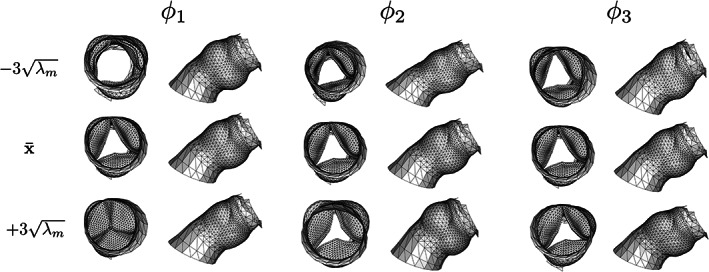
Visualization of the first three shape modes. The first shape mode represents the opening and closing of the aortic valve. The second shape mode seems to represent dilation of the base of the valve and the sinuses, the third mode seems to contain skewness/stretching in the sinuses

**FIGURE 6:**
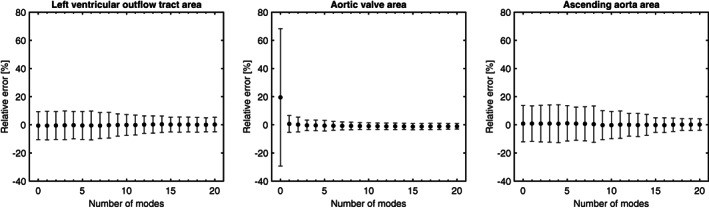
Mean absolute percent error of the LVOT, AV and AA areas as function of the number of modes. Error bars represent the mean absolute percent deviation. Steady improvements in left ventricular outflow tract area is observed between modes 7 and 11. Improvement in aortic valve area reconstruction is largest for the first mode. Improvement in ascending aorta area occurs gradually between modes 5 and 14

**FIGURE 7:**
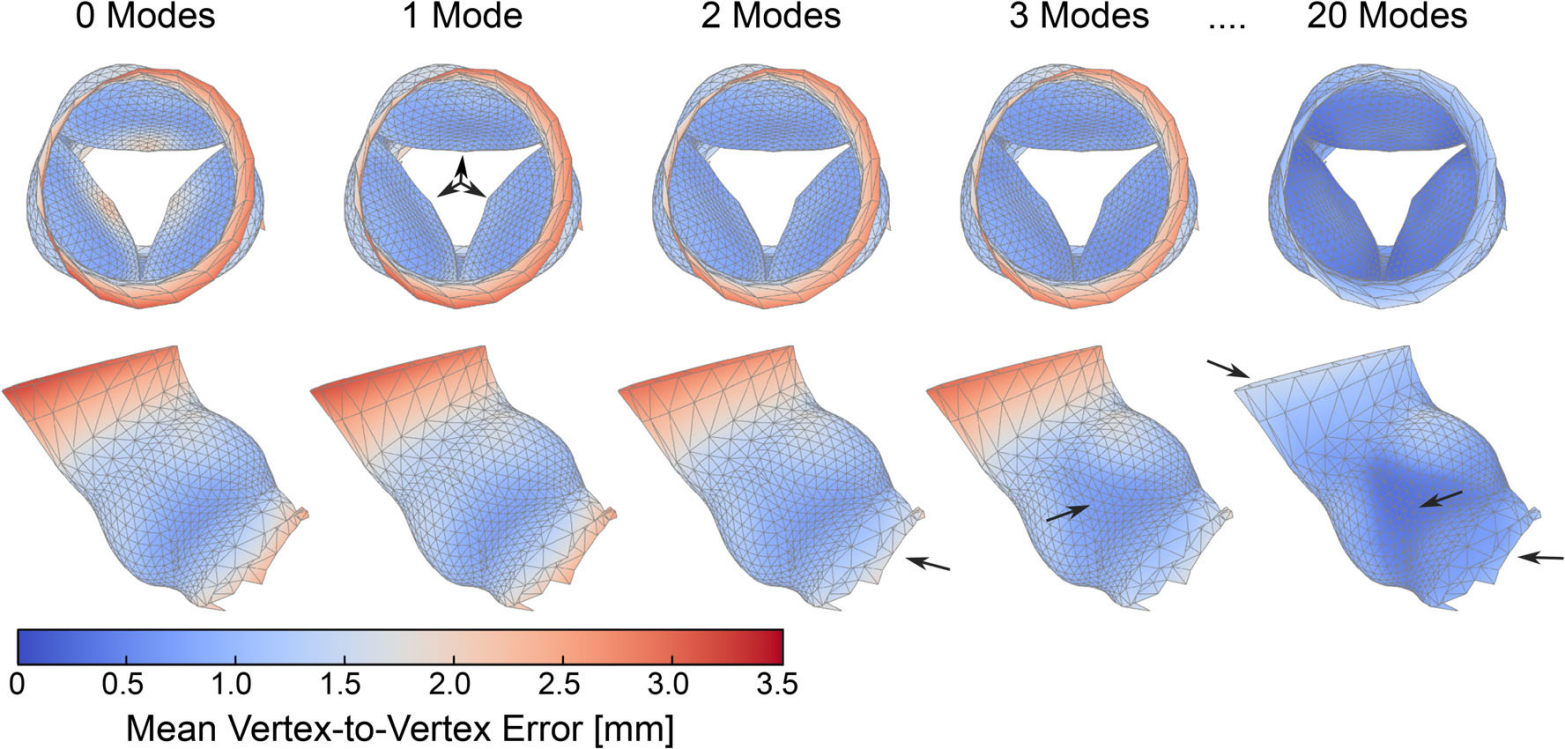
Mean vertex‐to‐vertex reconstruction error mapped onto the mean mesh ($ℳ\left(\bar{x},T\right)$). Top row: axial view, bottom row: side view. Mean vertex‐to‐vertex errors reduce with an increase in the number of modes. Main improvements with each added mode are highlighted with arrows. Vertices around the ascending aorta are poorly approximated with a low number of modes

In Figure 5 the mean mesh and the first three shape modes of the aortic valve are visualized. The first shape mode accounts for most of the variance (21%) and represents the opening and closure of the aortic valve. The valve is completely open at $-3\sqrt{\lambda_{1}}$, and completely closed at $3\sqrt{\lambda_{1}}$. The mean mesh ($\bar{x}$) features an aortic valve area of 170 mm^2^. The first shape mode reduces the reconstruction error of the aortic valve considerably (Figures 6 and 7). The second shape mode accounts for 14% of the variance, and seems to predominantly represent the size of the sinus/annulus region. This shape mode noticeably decreases the reconstruction error for vertices in the left ventricular outflow tract (Figure 7). The third mode accounts for 11% of the variance, and seems to mainly affect skewness of the sinus region. Improvements are mainly observed in the region where the valve is attached to the wall. Finally, Figure 7 also illustrates that with 20 shape modes, the mean vertex‐to‐vertex reconstruction error drops to below 1 mm. Furthermore, Figure 6 demonstrates that the error of the left ventricular outflow tract and ascending aorta areas gradually improve between 5 and 15 shape modes. No substantial improvement for these areas is observed beyond 15 modes.

**FIGURE 8:**
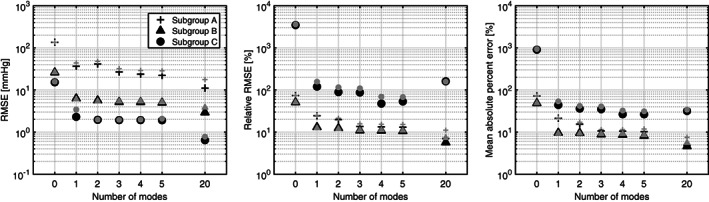
RMSE, relative RMSE, and mean absolute percent error (MAPE) of the pressure‐drop for subgroups A (+), B (▲), and C (•). Segmentation meshes were reconstructed with *N*_*m*_ = {1, 2, …5,20}. Substantial improvements are observed when including the first shape mode. Black: reconstruction performed with the SSM that is trained on all available segmentations. Gray: reconstruction based on a leave‐one‐out procedure: the segmented mesh is reconstructed with a SSM that is trained on all segmentations but the reconstructed segmentation

Figure 8 illustrates the compactness and generalizability of each model. The first three modes account for 46% of the variance. 80%, 90% and 95% of the variance in the training set is captured by 11, 19 and 29 modes respectively. Generalizability of the model levels out at 15 modes, meaning that the model is unlikely to generalize to unseen data with more than 15 shape modes.

### 3.3 CFD Performance of the shape model

To assess how many modes were required to build an accurate meta‐model, 2590 CFD simulations were performed, 74 meshes times five flow rates per mesh, times seven reconstructions (*N*_*m*_ = {0,1,2,3,4,5,20}). The computed pressure‐drop of each reconstruction was compared with the pressure‐drop that was computed on its segmentation counterpart at five flowrates (another 370 simulations), resulting in a pressure‐drop error. Pressure‐drop errors are expressed in RMSE, relative RMSE, and mean absolute percent errors (MAPE; Equations (17), (18), (19)).

**FIGURE 9:**
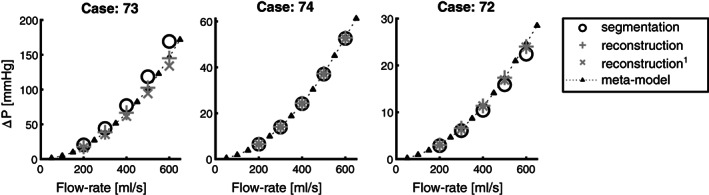
Pressure‐flow relationship for three typical cases in subgroups A, B, and C (from left to right). ○: CFD on segmentation mesh; +: CFD results for three‐mode reconstruction without leave‐one‐out procedure; **×**: CFD results for three‐mode reconstruction with leave‐one‐out procedure; ▲: meta‐model. Pressure‐flow relationships for all cases are illustrated in Figures 11‐13

Table 2 and Figure 9 demonstrate that the first shape mode—representing aortic valve opening/closing—substantially reduces the pressure‐drop error in all subgroups. RMSE values drop from 135.5 (subgroup A), 26.0 (subgroup B), and 15.2 mmHg (subgroup C), to 36.8, 6.3, and 2.3 mmHg respectively. These reductions may also be expressed in relative metrics. Relative RMSE values drop to 24.4%, 13.0%, and 119.9%. Mean absolute percent error values drop to 21.4%, 9.6%, and 44.1%. Although, RMSE, relative RMSE, and MAPE, still decrease when including more shape modes, improvements are more gradual. With a relative RMSE of 13.6% and 10.9%, and MAPE of 10.6% and 8.8%, improvements level out at three shape modes for subgroups A and B. Furthermore, it is shown that when segmentations are reconstructed with an excesssive number of modes (*N*_*m*_ = 20) the RMSE can drop to 11.1, 2.9, and 0.6 mmHg. Relative errors of 4.8% and 4.6% can be expected. Interestingly, subgroup C exhibits an increase in relative errors with respect to the model that is reconstructed with five shape modes, indicating that more shape modes do not necessarily improve pressure‐drop errors in the relative sense.

**TABLE 2:** RMSE, relative RMSE, and mean absolute percent error by *N*_*m*_ and subgroup

	RMSE [mmHg]			Relative RMSE [%]			MAPE [%]		
*N*_*m*_	A	B	C	A	B	C	A	B	C
0	135.5	26.0	15.2	73.4	50.8	3474.2	72.0	48.3	905.3
1	36.8	6.3	2.3	24.4	13.0	119.9	21.4	9.6	44.1
2	41.5	5.6	2.0	19.8	12.4	89.1	15.4	9.5	36.2
3	26.9	5.2	1.9	13.6	10.9	87.2	10.6	8.8	35.0
4	23.8	5.1	1.9	13.3	10.8	47.5	10.5	8.6	26.5
5	22.1	5.0	1.9	13.1	10.5	53.3	10.2	8.1	26.4
20	11.1	2.9	0.6	7.1	5.7	160.9	4.8	4.6	31.7

*Note*: All Results are based on reconstructions that were performed with the SSM that was trained on all segmentations, and correspond to the black symbols in Figure 9.

The reference pressure‐drop was computed by CFD on the segmentations.

Figure 9 demonstrates that leave‐one‐out results do not deviate much from the reconstructions with the full model, RMSE, relative RMSE, and MAPE follow the same trend as those of the full‐SSM.

**FIGURE 10:**
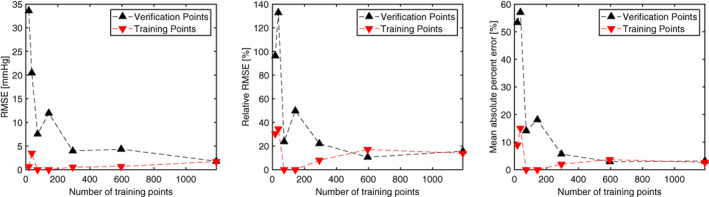
Meta‐model quality as function of the number of (successfully simulated) training points. Pressure‐drop errors follow from Equations (17), (18), (19)) with $\Delta {\hat{p}}_{i}$ the meta‐model approximation. CFD computed Pressure‐drops on the reconstructed meshes were used as the reference (Δ*p*_*i*_). Note: reference pressure‐drops were not used for training of the meta‐model

Typical pressure‐flow relationships for each subgroup and for segmentations and reconstructions (*N*_*m*_ = 3) are illustrated in Figure 10. Furthermore, results for all cases and subgroups can be found in Figures S11‐13. These figures support the observation that reconstruction seems to yield a fair approximation of the pressure‐flow relationship for subgroups A (Figure S11) and B (Figure S12). The pressure‐flow relationship is poorly approximated for several cases in subgroup C (Figure S13—cases: 15, 46, 47, and 51). The pressure‐drop errors do not seem to improve much beyond three shape modes. Therefore, a single meta‐model that takes into account the first three shape modes is generated.

### 3.4 Meta‐model performance

Seven meta‐models were build with a Genetic‐Aggregation response surface.^27^ Meta‐models were trained with 25, 50, 100, 200, 400, 800 and 1600 synthetic points/samples. Quality of the meta‐model depends on the number of available training samples (Figure 14). Relative RMSE and mean absolute percent errors of the verification points do not improve beyond 600 training points, and level out at approximately 11% and 3% respectively. The RMSE decreases to a value of 1.7 mmHg.

Moreover, Figure 14 demonstrates that two meta‐models exhibit RMSE, relative RMSE, and mean absolute percent errors of 0. That is, a perfect fit through all training points is obtained, suggesting that these models may have overfit the data. All other models show a non‐zero error. In general, the meta‐models with a low number of training points showed large errors on the verification points. However, errors on verification points substantially reduce when more training points (>300) were considered. This indicates that with a low number of training points, the (non‐linear) behavior of the pressure‐drop with respect to the shape coefficients is not adequately captured, that is, the meta‐model is of poor quality. Quality improves drastically beyond 300 training points, and yields an acceptable meta‐model that captures behavior of the pressure‐drop with respect to the shape coefficients well.

## 4 DISCUSSION

The pressure‐drop across the aortic valve is a key hemodynamic metric to evaluate the severity of aortic valve stenosis. The main aim of this work was to investigate whether a meta‐model can replace CFD simulations to find this pressure‐drop from segmented aortic valves. To achieve this, segmentation meshes of the aortic valve were parameterized by means of statistical shape modeling. Using a SSM, segmentation meshes were reconstructed by a limited number of shape modes and their corresponding coefficients. With the SSM a set of synthetic training meshes were generated. Consequently, CFD‐computed pressure‐drops of the synthetic meshes were used to train a meta‐model. The meta‐model replaces the compute‐intensive three‐dimensional CFD simulations by analytically relating three SSM shape modes, scaling, and flow rate, to the pressure‐drop. This meta‐model provides an estimate of the pressure‐flow relationship of a segmented aortic valve in real‐time.

The findings of this study illustrate that relevant geometrical variation of the aortic valve can be adequately captured by the SSM. The SSM captures the range of possible open/closed configurations of the valve. In particular, for subgroups A and B, the reconstruction and meta‐model results are close to the CFD results of the segmented mesh. On average, reconstruction introduces an error in pressure‐drop computations of only 10%, and further simplification to a meta‐model introduces an additional 3% error on average. Even though the first three shape modes account for only 46% of the variance, the essential geometrical features—relevant for pressure‐drop computations—seem to be adequately captured. One major cause of pressure‐drop errors for subgroups A and B is poor approximation of the AVA (Figure 15). This observation supports favoring physically relevant vertices through weighting of the minimization problem (Equation 9). Weighting lowers the reconstruction error for the vertices part of, or adjacent to the free cusp edge, and consequently it is more likely that an accurate estimate of the aortic valve orifice area is obtained. The aortic valve is more open for subgroup C, and the effect on the pressure‐drop is less pronounced. Hence, reconstruction errors in other regions will start to influence the pressure‐drop as well. For example, reconstruction errors for the sinus, left ventricular outflow tract, and aorta will become more important. Nevertheless, RMSE values indicate that the error is limited, and errors of 1.9 mmHg are expected for subgroup C.

For this study, a Genetic‐Aggregation meta‐model^27^ was trained that took into account just three shape modes. However, Table 2 suggests that with 20 modes, errors for subgroups A and B may further decrease to 4.8% and 4.6% respectively. Although not unfeasible, extensive computational costs are expected when the meta‐model is trained on such a high‐dimensional input space. We suggest that for such high‐dimensional models additional training points are sequentially added until the meta‐model is of sufficient quality.

The large number of publications in various bio‐medical fields proves that statistical shape modeling is a versatile and widely accepted technique to capture anatomic variation in the population. For example, it is extensively used for organ segmentation,^36^, ^37^, ^38^ to extract morphological bio‐markers of (diseased) organs,^39^, ^40^, ^41^ or for numerous orthopedic applications.^42^, ^43^, ^44^, ^45^, ^46^, ^47^ Although few, several studies—mainly in the field of orthopedics—attempted to relate SSM parameterized anatomical shape variation with Finite Element Analysis (FEA). These studies used SSM and FEA to: investigate the relationship between patellofemoral shape and function^48^; force‐displacement behavior of proximal femurs^49^; to investigate cervical spine loading,^50^ or for real‐time prediction of joint‐mechanics.^51^ Literature that combines SSM with FEA/CFD simulations is more scarce in the field of cardiovascular biomechanics. To our knowledge, only Khalafvand and colleagues used a SSM in combination with CFD simulations. They utilized a SSM‐driven simulation framework to systematically analyze blood flow in the left ventricle.^52^ However, they limited their simulations to five characteristic shapes, and did not attempt to build a cheap‐to‐evaluate meta‐model that approximates the simulation results over the entire shape space. Nevertheless, the work of Khalafvand et al, and our work demonstrate that the properties of SSM's can be exploited to yield simulation‐derived, physically relevant, hemodynamic metrics.

Although the aortic valve has a complex shape and function, various authors proposed simplified parametric models.^2^, ^53^, ^54^, ^55^ These parametric models facilitate parametric numerical simulations. For example, to study: collagen remodeling^54^; the effect of aortic valve geometry on peak‐stress,^2^ or bi‐cuspid geometry on ascending aorta hemodynamics.^56^ Such parametric models are particularly powerful for obtaining fundamental understanding of the involved physics, and for determining the most relevant physical parameters. However, the relationships that are found may not hold for each patient specifically. Therefore, there seems to be a shift towards using patient‐specific geometries as input for computational models.^57^, ^58^ In this study, an attempt was made to parameterize the patient‐specific geometries with a SSM. Fundamentally, the shape modes and shape coefficients have no physical meaning, which makes interpretation difficult. To aid interpretation, shape modes can be related to physical measures.^59^ In this study, the first shape mode seems to be both statistically and physically the most relevant. However, this may not always be the case. Hence, future work could use sensitivity analysis to select the most physically relevant shape modes.

### 4.1 Clinical applications

Pressure‐drops are indicative of aortic valve stenosis severity, and are particularly useful to determine the stenosis‐induced hemodynamic burden on the patient. Hence, the methodology that was proposed may be used to augment geometrical information from imaging systems with physiological, simulation‐derived metrics that are indicative of disease severity. For routine clinical use, such decision support systems need to be robust, easy to use, and computationally cheap. The meta‐model approach that is proposed would be particularly suitable for such a system.

The methodology that is proposed is also suitable for personalizing specific components of lumped‐parameter models. Lumped‐parameter models are popular in the cardiovascular research community to model the full‐body circulation. Heart‐valves in these lumped‐parameter models are generally described by orifice models that relate the pressure‐drop to the flow‐rate.^60^, ^61^ The meta‐model that is proposed finds this relationship from imaging data, in real‐time. Aortic valve segmentation, in combination with the meta‐model, may therefore be used to further personalize these lumped‐parameter models to more adequately predict the full‐system hemodynamics.

Moreover, it is noted that—clinically—subgroup B is the most interesting group. The aortic valve area is between 100 and 150 mm^2^, and the potential impact of the stenotic valve is not obvious. Subgroup C is clinically the least interesting, and the larger (relative) errors would not be problematic.

Finally, the authors would like to note that this is a purely numerical study, and clinical studies would be necessary to establish whether this approach yields complementary value in the clinic. Clinically, 4v^2^ is used to estimate the pressure‐drop over the valve.^35^ However, 4v^2^ represents the maximum pressure‐drop, and neglects pressure‐recovery, which may lead to overestimation of stenosis severity.^62^ Hence, this study aimed to predict the recovered pressure‐drop instead. Nevertheless, the same methodology could be used to predict the maximum pressure‐drop, and would be useful when moving towards clinical validation of the proposed methodology. Unfortunately, patient‐specific flow measurements were not available to the authors, and the methodology is patient‐specific with respect to geometry only.

### 4.2 Limitations

It was shown that the methodology that is proposed could help make CFD simulations more clinically accessible. Specific components of the methodology, such as establishing the required number of shape modes, and meta‐model training were validated. Other components of the methodology are less well validated. For example, (automatic) valve segmentation currently lacks a thorough and systematic validation. An idealized tri‐cuspid deformable template of the valve was used, which captured the overall feature of the valve, but details such as calcific deposits were not included. Nevertheless, Weese et al^15^ approximated the mean segmentation error on the valve leaflets to be around 0.47 mm, which is in the order of image resolution. Furthermore, the results in this study show, that accurate segmentation in the proximity of the cusp's free edges is desirable. A more thorough understanding of segmentation error may be obtained by having (clinical) experts manually create or edit “ground‐truth” valve segmentations as a reference, for example, see the work by Ecabert et al^16^ and Pouch et al^18^ for examples. In addition, the authors would like to note that, although accurate segmentation is necessary, the meta‐model approach allows the propagation of segmentation uncertainties to uncertainties in output parameters. That is, uncertainties in valve segmentations could be related to uncertainties in shape mode coefficients. In turn, uncertainties in the shape mode coefficients could be used to obtain an estimate of the pressure‐drop, augmented with its respective confidence interval.

As a consequence of the idealized template mesh, calcifications and bi‐cuspid valves were not represented in the SSM. SSM worked well with Computed Tomography images, but images from 3D transesophageal echocardiography lack the spatial resolution for adequate automatic segmentations, and often need manual corrections. These manual corrections would add additional variation in a SSM training set, yielding a less compact and general SSM. Moreover, the SSM requires segmentation models with consistent topology, which may not always be available.

The CFD models in this study represent a time‐averaged, fully developed, (turbulent) flow. However, flow through the aortic valve is pulsatile in nature, and may not reach the fully developed turbulent state. However, it has been shown that for pressure‐drops above 10 mmHg, the steady‐state assumption gives a reasonable estimate for the peak‐systolic pressure‐drop.^13^ Furthermore, it is noted that the proposed methodology is not limited to steady‐state simulations, and simulation results of more realistic, time‐dependent, and/or fluid‐structure interaction simulations could be used in future studies.

### 4.3 Conclusion

This study evaluated the performance of a SSM‐based meta‐model. SSM is shown to adequately capture aortic valve shape variation. The shape coefficients of the SSM are successfully used to train a meta‐model that analytically relates the shape coefficients to the pressure‐flow relationship. Moreover, it is shown that the SSM‐based meta‐model provides an acceptable assumption of the pressure‐flow relationship, and given adequate training data, is a viable real‐time alternative to 3D CFD simulations of the aortic valve.

## Supporting information


**Appendix.**
