# Supplementary material for: Combining statistical shape modeling, CFD, and meta‐modeling to approximate the patient‐specific pressure‐drop across the aortic valve in real‐time
Source: Int J Numer Method Biomed Eng. 2020 Sep 13;36(10):e3387. doi: 10.1002/cnm.3387 (PMC7583374; doi:10.1002/cnm.3387)
Supplement: Supplementary file 1 — Appendix. [file CNM-36-e3387-s001.pdf]

1 | APPENDIX

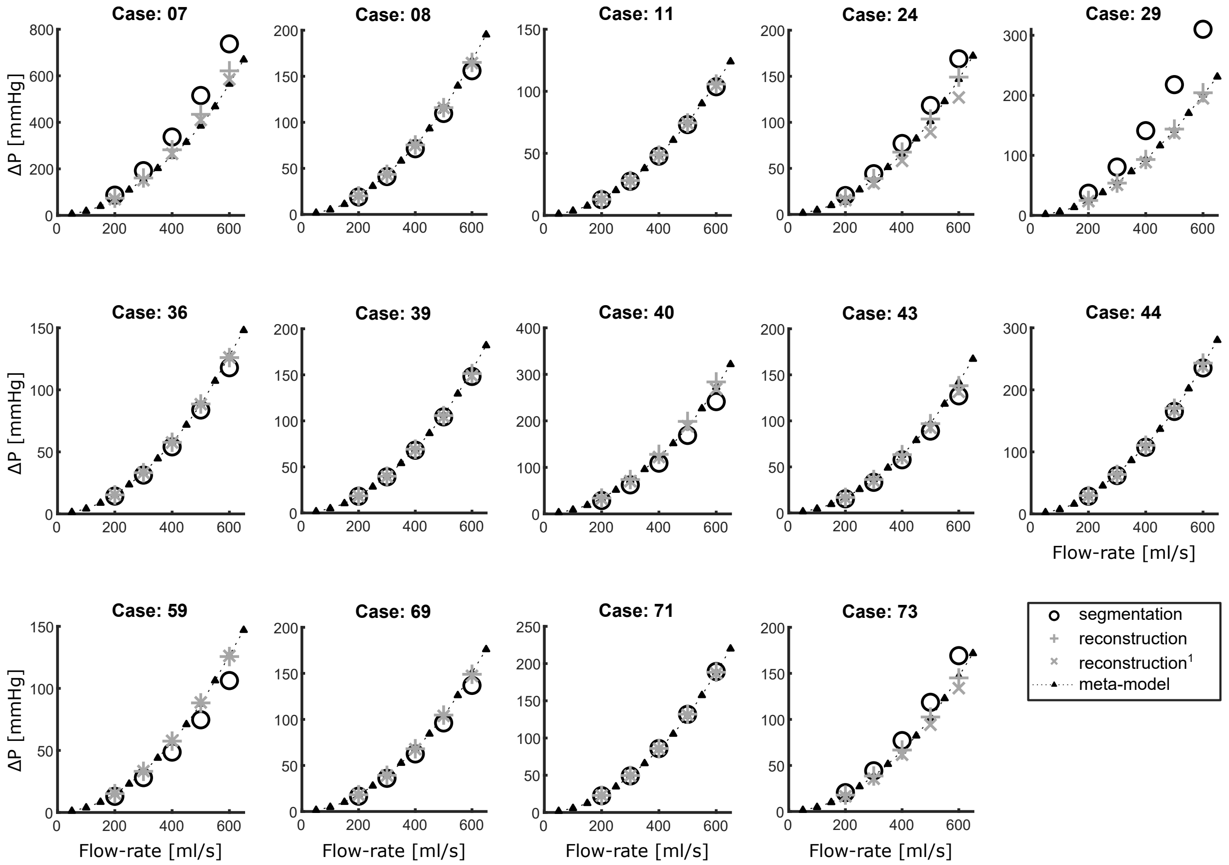

FIGURE 11 [SUPPLEMENTARY] Subgroup A: cases with AVA ≤ 1.0 cm^2. ○: CFD segmentation mesh; +: CFD 3-mode reconstruction without leave-one-out procedure; ×[1]: CFD 3-mode reconstruction with leave-one-out procedure; ▲: Meta-model

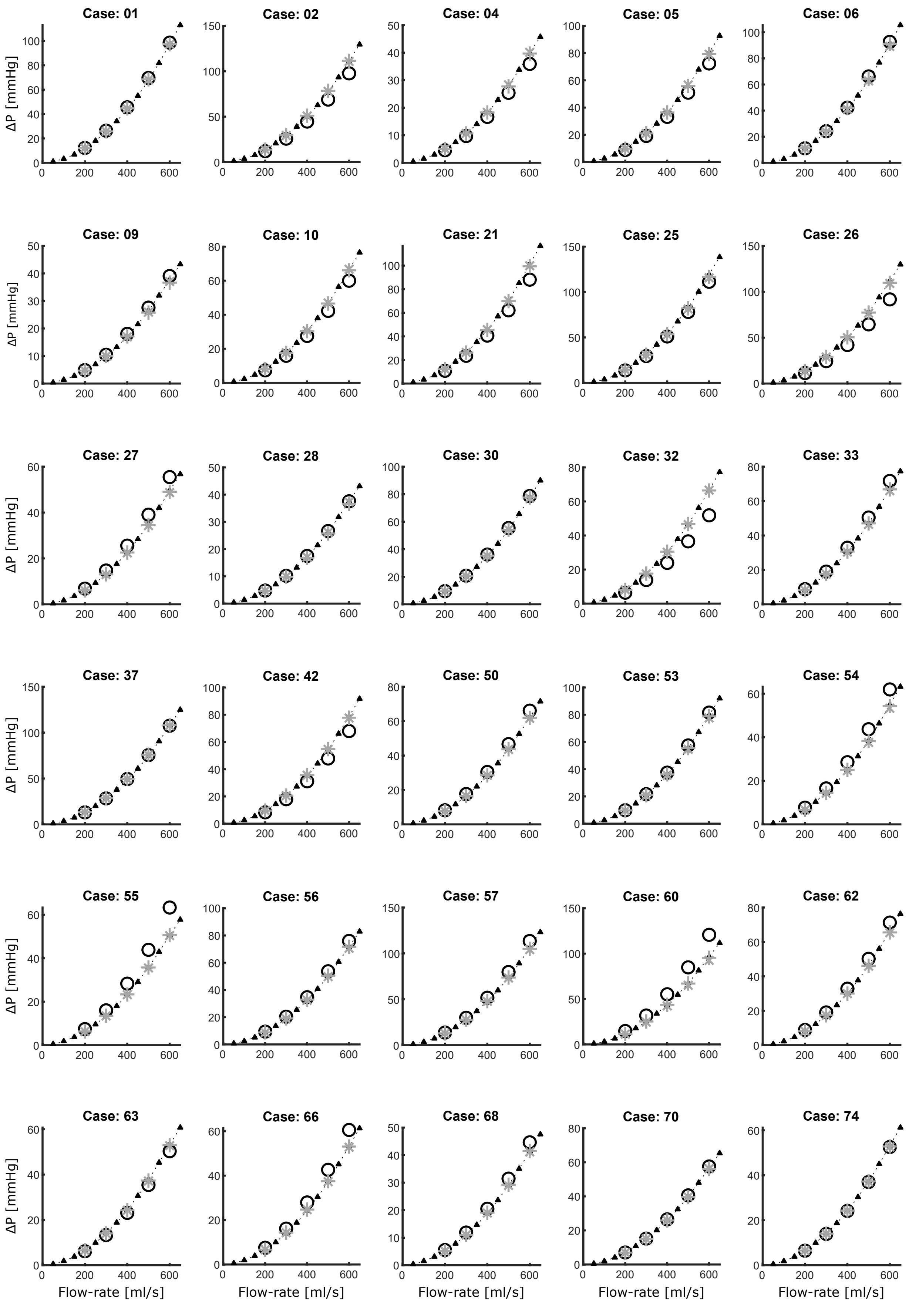

FIGURE 12 [SUPPLEMENTARY] Subgroup B: cases with $1.0 < \text{AVA} \leq 1.5\ \text{cm}^2$. ○: CFD segmentation mesh; +: CFD 3-mode reconstruction without leave-one-out procedure; ×: CFD 3-mode reconstruction with leave-one-out procedure; ▲: Meta-model

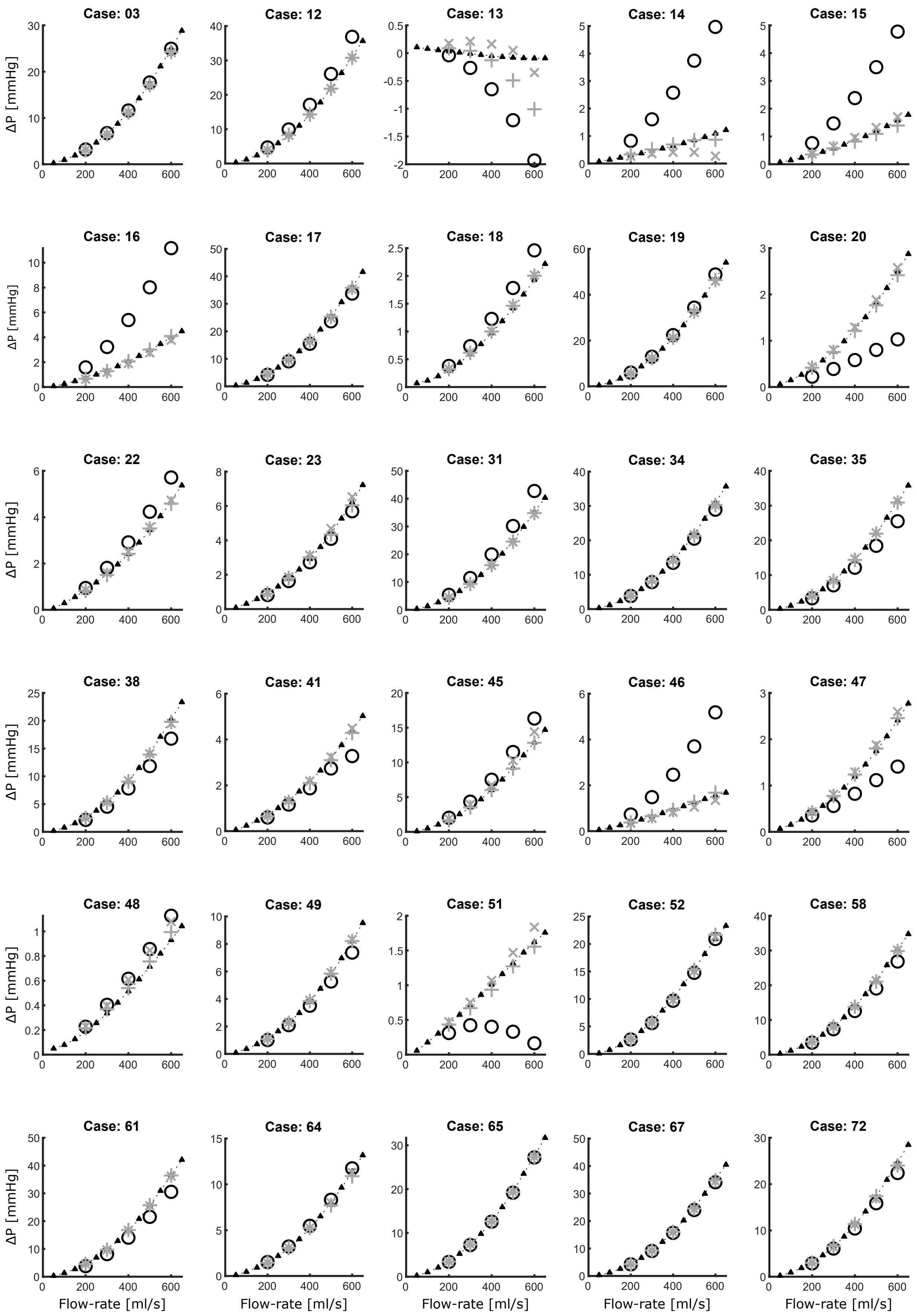

FIGURE 13 [SUPPLEMENTARY] Subgroup C: cases with AVA > $1.5\ cm^2$. ○: CFD segmentation mesh; +: CFD 3-mode reconstruction without leave-one-out procedure; ×: CFD 3-mode reconstruction with leave-one-out procedure; ▲: Meta-model

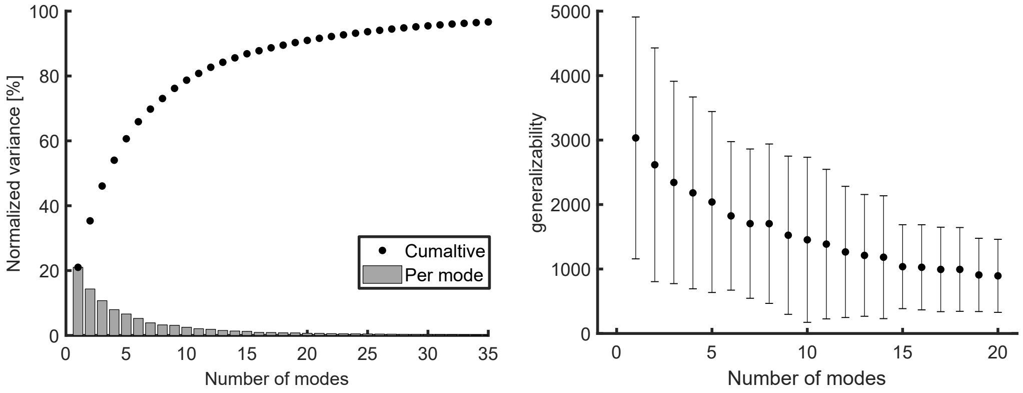

FIGURE 14 [SUPPLEMENTARY] Compactness (left) and generalization ability (right) as function of the number of modes. The first three shape modes capture 46% of the variance.

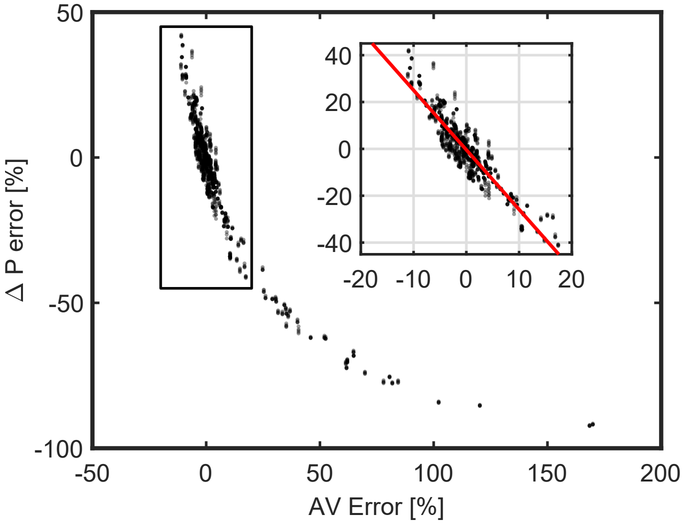

FIGURE 15 [SUPPLEMENTARY] Pressure-drop error as function of the AVA reconstruction error. Subgroups A and B (AVA $\leq$ 150mm^2) were considered, and CFD results of all their corresponding reconstructions ($N_m = 0, 1, ..., 5, 20$) are used (1540 simulations). A linear model is fitted on the aortic valve error interval $[-20, 20]$. A slope of -2.54 is found ($R^2 = 0.838$), and indicates that with every percent error in AVA reconstruction, 2.5 % error in ΔP can be expected.
